# Artificial intelligence in trigeminal neuralgia: trigeminal nerve segmentation and neurovascular conflict detection: a systematic review and meta-analysis

**DOI:** 10.1007/s10143-026-04486-5

**Published:** 2026-09-14

**Authors:** Mohammadamin Sabbagh Alvani, Ibrahim Mohammadzadeh, Bardia Hajikarimloo, Melika Javani, Shahin Mohammadzadeh, Ali Mortezaei, Amirhossein Zare, Farzad Fayedeh, Sai Sanikommu, Amirreza Khalaji, Mohammad Amin Habibi, Adam A. Dmytriw, Farzan Fahim, Seyed Ali Mousavinejad, Ahmet Günkan, Qais Alrashidi, Hamid Borghei-Razavi

**Affiliations:** 1 Skull Base Research Center, Loghman-Hakim Hospital, Shahid Beheshti University of Medical Sciences, Tehran, Iran; 2 Department of Neurological Surgery, University of Virginia, Charlottesville, VA USA; 3 Shahid Beheshti University of Medical Sciences, Tehran, Tehran Islamic Republic of Iran; 4 Student Research Committee, Gonabad University of Medical Sciences, Gonabad, Iran; 5 Neurology Department, Tehran university of medical sciences, Tehran, Iran; 6 Student Research Comittee, Birjand University of medical sciences, Birjand, Iran; 7 Department of Neurological Surgery, Radiology, Neurosciences, Pharmacology, University of Miami School of Medicine, Miami, FL USA; 8 Faculty of Medicine, Tabriz University of Medical Sciences, Tabriz, Iran; 9 Department of Neurosurgery, Shariati Hospital, Tehran University of Medical Sciences, Tehran, Iran; 10 Neuroendovascular Program, Massachusetts General Hospital & Brigham and Women’s Hospital, Harvard Medical School, Boston, MA USA; 11 Division of Vascular and Interventional Radiology, Department of Radiology and Imaging Sciences, University of Arizona, Tucson, AZ USA; 12 Department of Neurological Surgery, Pauline Braathen Neurological Center, Cleveland Clinic Florida, Weston, FL USA; 13 Cleveland Clinic Lerner College of Medicine of Case Western Reserve University, Cleveland, OH USA; 14 Director of Brain Tumor and Pituitary Center, Neuroscience Institute, Cleveland Clinic Lerner College of Medicine of Case Western Reserve University, Cleveland Clinic Florida Region, 2950 Cleveland Clinic Blvd, Weston, FL 33331 USA

**Keywords:** Trigeminal neuralgia, Artificial intelligence, Deep learning, Machine learning, Diagnostic accuracy, Meta-analysis.

## Abstract

Trigeminal neuralgia (TN) is a debilitating condition characterized by severe, episodic facial pain, often caused by neurovascular conflict (NVC) at the trigeminal nerve root. Early and accurate diagnosis of TN is crucial for effective management, with microvascular decompression (MVD) being a common surgical treatment. While MRI plays a key role in detecting NVC, its subjective nature limits diagnostic precision. Recent advances in artificial intelligence (AI) offer promising tools for enhancing diagnostic accuracy. This study evaluates the potential of deep learning (DL) and machine learning (ML) models in improving TN diagnosis through automated segmentation and NVC detection in MRI scans. A comprehensive search was conducted across PubMed, Scopus, Embase, Web of Science, and the Cochrane Library to identify studies utilizing AI models in TN diagnosis. Studies assessing sensitivity, specificity, accuracy, and AUC were included in the meta-analysis. A total of 5 studies met the inclusion criteria, encompassing 577 patients with confirmed TN. The primary focus was on evaluating the performance of AI models in segmenting the trigeminal nerve and identifying neurovascular conflicts. The reference standard or target criterion used to define correct model performance was also extracted and considered, including expert imaging-based annotation, clinical-radiological diagnosis, and intraoperative confirmation where available. The pooled sensitivity of AI models in detecting TN was 71% (95% CI: 65–76%), with a specificity of 92% (95% CI: 88–94%). The positive diagnostic likelihood ratio (DLR) was 8.5 (95% CI: 5.75–12.56), and the negative DLR was 0.32 (95% CI: 0.26–0.38). The diagnostic odds ratio (DOR) was 26.71 (95% CI: 16.38–43.56), and the area under the curve (AUC) reached 0.91 (95% CI: 0.88–0.93). These findings demonstrate the high diagnostic accuracy of AI models, particularly in identifying NVC and improving preoperative planning for TN patients. AI models, particularly DL-based approaches, show promising diagnostic performance in the segmentation of the trigeminal nerve and detection of NVC. The high specificity and diagnostic odds ratio suggest that AI can play a critical role in enhancing clinical decision-making and improving the accuracy of TN diagnosis, ultimately aiding in better treatment planning for patients.

**Supplementary Information:**

The online version contains supplementary material available at https://doi.org/10.1007/s10143-026-04486-5.

## Introduction

Trigeminal neuralgia (TN), among the most prevalent forms of neuropathic pain, is characterized by intense, episodic, shock-like facial pain that can be triggered by innocuous stimuli and routine orofacial activities, including speaking, chewing, and eating. These painful attacks substantially impair daily functioning and are associated with increased anxiety, depression, and compromised sleep [1, 2]. Epidemiological studies indicate that TN, typically unilateral, has a prevalence range of 0.03–0.3% and an incidence rate of about 4 to 5.5 per 100,000 person-years, with a higher occurrence in females [3–5]. Trigeminal neuralgia is classified as classic, secondary, or idiopathic. The classic type is most often attributed to neurovascular conflict (NVC), typically involving compression by the superior cerebellar artery. Secondary TN is less common and is caused by identifiable structural or neurological conditions, such as cerebellopontine angle tumors, vascular malformations, or demyelinating plaques [6, 7].

As mentioned, NVC includes a range of conflicts from simple contacts of the arteries and veins within the nerve root entry zone to extreme compressions, resulting in atrophic or displacement alterations of the trigeminal nerve [8]. While NVC is the leading cause of classic TN and can trigger nerve damage and pain signals, it is not a definitive diagnostic marker [9]. Many asymptomatic people have this compression, while some TN patients lack it, making its presence or absence insufficient to confirm or rule out the condition, hence a beneficial suggestive tool rather than a definite diagnostic one [7, 9]. Preoperative high-resolution MRI is an essential component in surgical planning for the surgical treatment of trigeminal neuralgia. MRI can effectively identify neurovascular contact and rules out secondary etiologies and provides substantial evidence to evaluate the diagnosis of classic TN and the appropriateness of microvascular decompression (MVD) as a corrective intervention [10, 11]. Several MRI-based modalities, including magnetic resonance angiography (MRA), T2-weighted imaging, and 3D MRI sequences, have shown promising but variable diagnostic performance for detecting NVC in TN. However, MRI interpretation remains observer-dependent; in one real-world study, radiologists detected surgically confirmed arterial compression with a sensitivity of 50.9% and specificity of 81.8%, whereas neurosurgical review showed a sensitivity of 87.7% and specificity of 72.7% 15. Therefore, MRI is an essential but not definitive tool for evaluating NVC and planning MVD [12–15].

Artificial intelligence (AI) systems using routine MRI scans could help clinicians make better treatment decisions for TN. Radiomics-based AI models can spot imaging patterns that radiologists might miss. They analyze features like statistics, shape, and texture, and once trained, can process scans and deliver predictions in just seconds, making the diagnosis, patient classification, and treatment decisions more efficient, precise, and time-saving [16–18]. Recent progress in AI has made it possible to achieve reliable, fully automated segmentation of various anatomical structures across different imaging modalities, particularly through DL algorithms based on neural networks [19]. Through complete automation of segmentation and analysis, reader disagreement can be significantly reduced. The significance of this AI-supported method in important clinical decision-making has already been shown repeatedly across various fields [20–24].

In this study, we aimed to investigate the application of AI, particularly DL–based methods, in the segmentation of the trigeminal nerve and detection of NVC on MRI in patients with TN. Through a systematic review and meta-analysis, we evaluated the methodologies, performance, and clinical relevance of AI-driven approaches in identifying anatomical features critical for diagnosis and surgical planning. The primary goal was to determine how these AI-based models can support clinical decision-making by providing accurate, reproducible, and objective assessments of the trigeminal nerve and its surrounding vasculature. This review focuses specifically on the role of AI in nerve segmentation and NVC detection, rather than functional imaging or other applications of AI in TN.

## Methods

### Study design

This systematic review and meta-analysis aimed to assess the clinical application of AI-based approaches for trigeminal nerve segmentation and neurovascular conflict (NVC) detection in patients with trigeminal neuralgia (TN). The study was conducted in accordance with the Preferred Reporting Items for Systematic Reviews and Meta-Analyses (PRISMA) guidelines to ensure a standardized and transparent methodology [25].

### Search strategy

From their inception through May 2025, we conducted a systematic search in PubMed, Embase, Scopus, the Cochrane Library, and Web of Science without limiting the type of publication. To broaden coverage, we also screened the first 100 results on Google Scholar. The search strategy combined the terms (“Trigeminal neuralgia” OR “TN”) AND (“artificial intelligence” OR “AI” OR “machine learning” OR “deep learning”). This query was applied across all databases, with MeSH terms in PubMed and Emtree terms in Embase incorporated alongside related keywords to optimize retrieval for each source. The complete search syntax is provided in the supplementary file in Table S1.

### Inclusion and exclusion criteria

We focused on studies that examined patients with TN using MRI to evaluate trigeminal nerve segmentation and/or NVC detection with AI-based methods. Eligible studies were original research articles, including cohort studies, randomized trials, and case series. Case series were considered only when they included at least 10 patients and provided sufficient quantitative data for extraction.

Studies were excluded if they were non-original articles, including reviews, editorials, commentaries, or conference abstracts; were not written in English; included fewer than 10 patients; lacked sufficient data for extraction; or reported overlapping datasets.

### Study selection and data extraction

Following the database search, duplicate records were first removed, and the remaining studies were screened independently by two reviewers at the title and abstract level. Articles that appeared relevant were then assessed in full text. Any disagreements in selection were resolved with input from a third reviewer. All references were managed in EndNote version 21 throughout the screening process. For data extraction, two reviewers worked independently with a standardized Microsoft Excel template, extracting the defined study variables. The extracted study characteristics included publication year, geographic location, study design, and patient demographics. Information on AI approaches was collected, detailing the type of AI used (ML or DL), the specific classifiers applied, and the validation methods employed. Reported outcomes encompassed performance metrics such as AUC, accuracy, sensitivity, specificity, Dice similarity coefficient, Jaccard index, precision, and other relevant measures related to diagnostic or segmentation performance. In addition, we extracted the target outcome predicted by each model and the reference standard used to define correct versus incorrect predictions. These reference standards included expert/manual imaging annotation, neuroradiological identification of neurovascular conflict, clinical-radiological diagnosis, symptom laterality, and intraoperative confirmation when available.

### Quality assessment

The methodological quality and risk of bias of the included studies were evaluated using the Prediction model Risk Of Bias Assessment Tool (PROBAST) [26]. This tool assesses the risk of bias across four key domains: Participants, Predictors, Outcome, and Analysis.

- Participants: This domain evaluates the characteristics of the study participants and the process used for their selectionParticipants: This domain evaluates the characteristics of the study participants and the process used for their selection.
- Predictors: This domain focuses on how the predictors (independent variables) are defined, measured, and applied in the modelPredictors: This domain focuses on how the predictors (independent variables) are defined, measured, and applied in the model.
- Outcome: This domain assesses the outcome measures (dependent variables) used in the study, evaluating their accuracy, validity, and reliabilityOutcome: This domain assesses the outcome measures (dependent variables) used in the study, evaluating their accuracy, validity, and reliability.
- Analysis: This domain evaluates the statistical methods employed in model development, including how potential confounders were controlled and how missing data were handledAnalysis: This domain evaluates the statistical methods employed in model development, including how potential confounders were controlled and how missing data were handled.

Each domain is evaluated using specific signaling questions to determine the risk of bias, categorized as low, high, or unclear risk. In cases of disagreement between the two primary reviewers, a third reviewer was consulted to resolve any conflicts.

Studies were classified as having an overall high risk of bias if at least one domain was rated as high risk. Studies with low or unclear risk in all domains were considered to have an overall low risk of bias.

### Statistical analysis

The statistical analysis was conducted using STATA version 17 with the MIDAS module. The key diagnostic metrics, including true positives, true negatives, false positives, and false negatives, were calculated based on the reported sensitivity and specificity of each eligible AI-based model in the included studies. The primary objective of this review was to assess the diagnostic accuracy of eligible AI-based models and compare their relative performance, while distinguishing conventional ML classifiers from DL architectures when applicable. A meta-analysis was performed to pool estimates for sensitivity, specificity, positive likelihood ratios (DLR+), negative likelihood ratios (DLR-), diagnostic odds ratio (DOR), diagnostic score, and the receiver operating characteristic (ROC) curve. This analysis aimed to identify the most effective ML algorithm from each study based on the highest reported performance metrics. The approach was used to estimate the potential of AI-based models in predicting outcomes and to provide a benchmark for comparison with traditional clinical tools. Given the variability in target criteria across the included studies, we categorized the extracted outcomes according to the reference standard used for model training or evaluation. Segmentation-based outcomes, such as Dice coefficient or Jaccard index, were distinguished from diagnostic classification outcomes, such as identification of symptomatic trigeminal nerves or neurovascular conflict. Diagnostic meta-analysis was restricted to outcomes for which binary diagnostic performance measures could be derived. The type of reference standard was considered as a source of methodological heterogeneity. Because of the small number of included studies, formal subgroup meta-analysis or meta-regression according to reference standard was not statistically reliable; therefore, this issue was addressed through structured descriptive comparison and cautious interpretation of pooled estimates.

## Results

### Study selection process

**Fig. 1:**
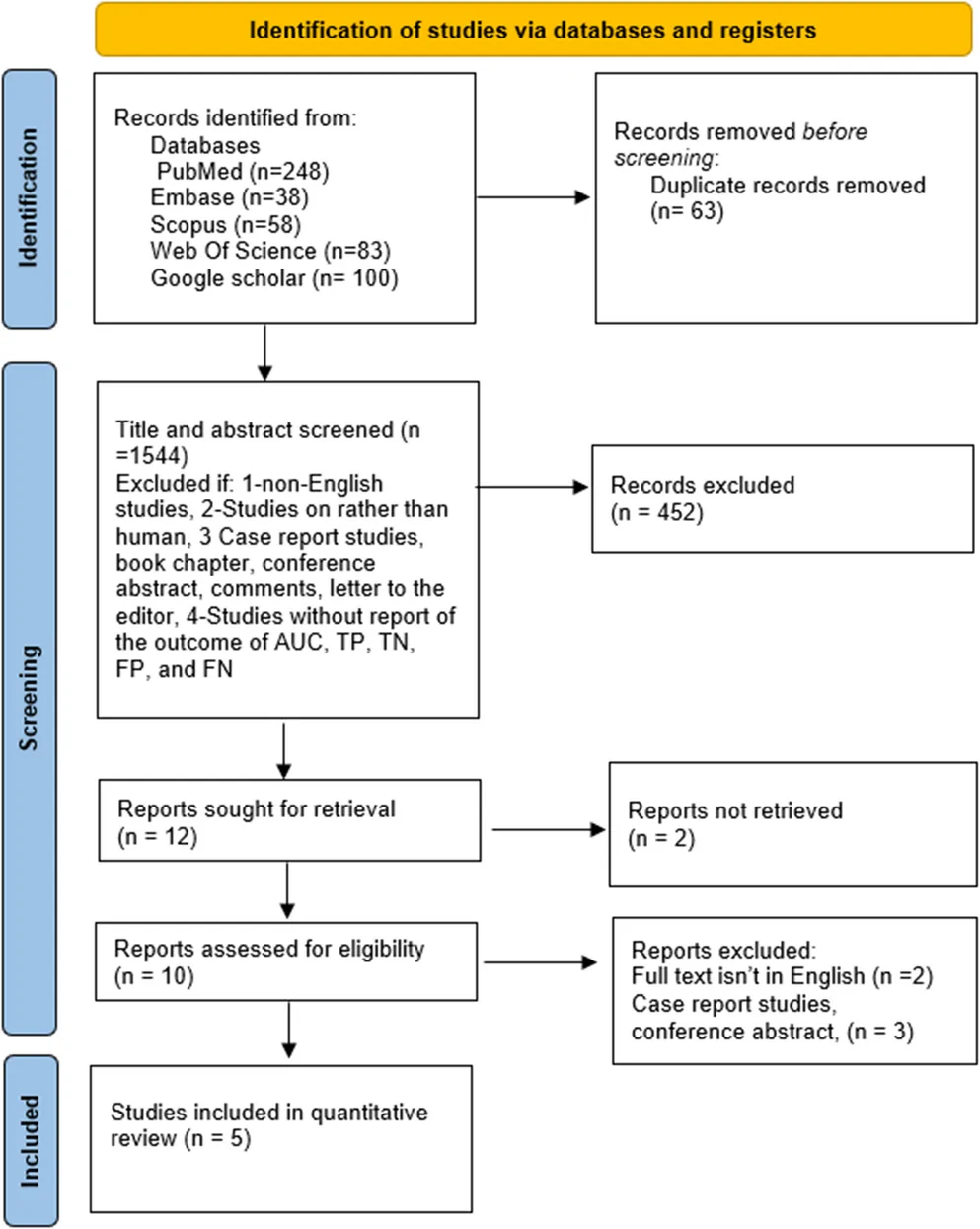
PRISMA flowchart of the study selection process

A total of 1544 records were identified from databases. After removing 63 duplicates, 1481 records remained. Following title and abstract screening, 452 records were excluded based on predefined criteria, leaving 1030 records for full-text review. 12 reports were sought, after assessing full texts, 3 studies were excluded due to language and conference abstracts without sufficient extractable data, resulting in 5 studies included in the final quantitative review (Fig. 1).

### Baseline characteristics

A total of 577 patients were included across 5 studies, with a mean age of 53.86 years. Among the participants, 68.02% were female. The studies varied in follow-up duration, with the mean follow-up period being 34.5 months (Tables 1 and 2).

**Table 1:** Demographic and characteristics of inclusion studies

Author/Year	Type of study	Country	Number of patients in train/test group	Type of TN studied (CTN, ITN, PTN, STN)	Diagnostic criteria	Mean age/female%	Inclusion criteria	Exclusion criteria	Differentiation of symptomatic vs. asymptomatic nerves
Wu et al.^1^./2025 [27]	Retrospective	China	93	CTN (28), ITN (22)	Clinical (ICHD-3) + Surgical confirmation of NVC + MRI	53/76	Primary TN, refractory to treatment, unilateral, diagnosed by ICHD-3	Bilateral TN, secondary TN, abnormal findings on MRI	NA
Lin et al.^2^./2021 [23]	Retrospective	China	50	Primary TN	Clinical diagnosis of TN + MRA imaging; segmentation validated against neurosurgeon-labeled ground truth	NA	TN patients undergoing MRA of root entry zone	NA	Yes, segmentation allowed visualization of compressed vs. non-compressed nerves in NVC
Halbert-Elliott et al.^3^/2025 [28]	Retrospective	USA	50	Classical TN, classified by Burchiel (Type 1 = 66%, Type 2 = 34%)	Clinical diagnosis + MRI-based neurovascular contact; confirmed intraoperatively (MVD)	59.1/60	Patients with TN undergoing MVD with pre-op high-resolution CISS MRI	Prior MVD, lack of high-res MRI, unusable imaging	Yes (both sides segmented per patient)
Zhang et al.^4^./2023 [22]	Retrospective	China	187	(TN patients + healthy controls, full TN root and branches segmentation	Clinical TN criteria (per ICHD/EAN) + MRI-based confirmation	55.45/61	TN patients undergoing MR neurography; healthy volunteers	Poor-quality MRI; segmentation mismatch with original coordinates	Yes (patients vs. healthy controls)
Hsu et al.^5^./2025 [24]	Prospective	Taiwan/USA/China	197	Clinical diagnosis + MRI-based confirmation; manual segmentation by neuroradiologists	Clinical diagnosis + MRI-based confirmation; manual segmentation by neuroradiologists	47.9/81.72	TN patients with MRI; healthy controls with high-quality MRI	Poor-quality MRI, incomplete labeling	Yes (HC vs. TN groups)

1. Wu M, Qiu J, Chen Y, Jiang X. Stratifying trigeminal neuralgia and characterizing an abnormal property of brain functional organization: a resting-state fMRI and machine learning study. Journal of Neurosurgery 2025;143(1):74-82.

2. Lin J, Mou L, Yan Q, et al. Automated segmentation of trigeminal nerve and cerebrovasculature in MR-angiography images by deep learning. Frontiers in Neuroscience 2021; 15:744967.

3. Halbert-Elliott KM, Xie ME, Dong B, et al. Deep learning–based segmentation of the trigeminal nerve and surrounding vasculature in trigeminal neuralgia. Journal of Neurosurgery 2025;1(aop):1-9.

4. Zhang C, Li M, Luo Z, et al. Deep learning-driven MRI trigeminal nerve segmentation with SEVB-net. Frontiers in Neuroscience 2023; 17:1265032.

5. Hsu L-M, Wang S, Chang S-W, et al. Automatic Segmentation of the Cisternal Segment of Trigeminal Nerve on MRI Using Deep Learning. International Journal of Biomedical Imaging 2025; 2025(1):6694599.

**Table 2:** ML models characteristics and performance metrics

Author/Year	Validation	Type of reference Sequence	Input characteristics	Selected features	Method of radiomics	Type of feature	Type of TN studied (CTN, ITN, PTN, STN)	Diagnostic criteria	Differentiation of symptomatic vs. asymptomatic nerves	ML algorithm
Wu et al.^1^./2025 [27]	10-fold cross-validation	Resting-state fMRI (BOLD), T1-weighted 3D MRI	Functional connectivity (rs-fMRI indices), fALFF, VAS scores	Recursive Feature Elimination (RFE) selected FC/fALFF indices	Automated	Functional connectivity indices + fALFF	CTN (28), ITN (22)	Clinical (ICHD-3) + Surgical confirmation of NVC + MRI	NA	SVM (RBF), RF, LR, kNN, GNB
Lin et al.^2^./2021 [23]	5-fold cross-validation	3D Magnetic Resonance Angiography (MRA)	Imaging features from MRA volumes (brainstem, cerebrovasculature, trigeminal nerve)	Multi-scale features with Res2Block + 3D U-Net refinement	Automated	Imaging-based segmentation features (voxel-level, multi-scale deep features)	Primary TN	Clinical diagnosis of TN + MRA imaging; segmentation validated against neurosurgeon-labeled ground truth	Yes, segmentation allowed visualization of compressed vs. non-compressed nerves in NVC	3D DL framework, 3D U-Net, V-Net, Uception, AnatomyNet, CS2-Net
Halbert-Elliott et al.^3^/2025 [28]	Single train/test split	High-resolution CISS MRI	Imaging features (MRI voxels, radiomics-like deep features)	Segmentation-based features (surface area of neurovascular contact, distance to conflict)	Automated	Imaging (voxel-based, geometric measurements)	Classical TN, classified by Burchiel (Type 1 = 66%, Type 2 = 34%)	Clinical diagnosis + MRI-based neurovascular contact; confirmed intraoperatively (MVD)	Yes (both sides segmented per patient)	ResNeXt50, ResNeXt50, DenseNet121, VGG16, IncV3, SE-RN50
Zhang et al.^4^./2023 [22]	Train/test split	3D T2 MRI (multi-scanner, MR neurography)	Imaging (voxel-based MRI features)	Segmentation volumes (root + three branches of TN)	Automated	Imaging (voxel-based)	(TN patients + healthy controls, full TN root and branches segmentation	Clinical TN criteria (per ICHD/EAN) + MRI-based confirmation	Yes (patients vs. healthy controls)	V-Net, nnUNet, SEVB-Net,
Hsu et al.^5^./2025 [24]	80/20 train-validation split	3D T1-weighted MRI (MP-RAGE)	Imaging (structural MRI voxels)	Volumetric + voxel-based segmentation (cisternal TN)	Automated	Imaging (voxel-level)	Clinical diagnosis + MRI-based confirmation; manual segmentation by neuroradiologists	Clinical diagnosis + MRI-based confirmation; manual segmentation by neuroradiologists	Yes (HC vs. TN groups)	3D U-Net

1. Wu M, Qiu J, Chen Y, Jiang X. Stratifying trigeminal neuralgia and characterizing an abnormal property of brain functional organization: a resting-state fMRI and machine learning study. Journal of Neurosurgery 2025;143(1):74-82.

2. Lin J, Mou L, Yan Q, et al. Automated segmentation of trigeminal nerve and cerebrovasculature in MR-angiography images by deep learning. Frontiers in Neuroscience 2021; 15:744967.

3. Halbert-Elliott KM, Xie ME, Dong B, et al. Deep learning–based segmentation of the trigeminal nerve and surrounding vasculature in trigeminal neuralgia. Journal of Neurosurgery 2025;1(aop):1-9.

4. Zhang C, Li M, Luo Z, et al. Deep learning-driven MRI trigeminal nerve segmentation with SEVB-net. Frontiers in Neuroscience 2023; 17:1265032.

5. Hsu L-M, Wang S, Chang S-W, et al. Automatic Segmentation of the Cisternal Segment of Trigeminal Nerve on MRI Using Deep Learning. International Journal of Biomedical Imaging 2025; 2025(1):6694599.

Geographically, the majority of studies were conducted in China, representing 60% (3 out of 5), while the remaining studies were conducted in the USA and a combined Taiwan/USA/China cohort, each accounting for 20% (1 out of 5) of the studies (Tables 1 and 2)**.**

**Fig. 2:**
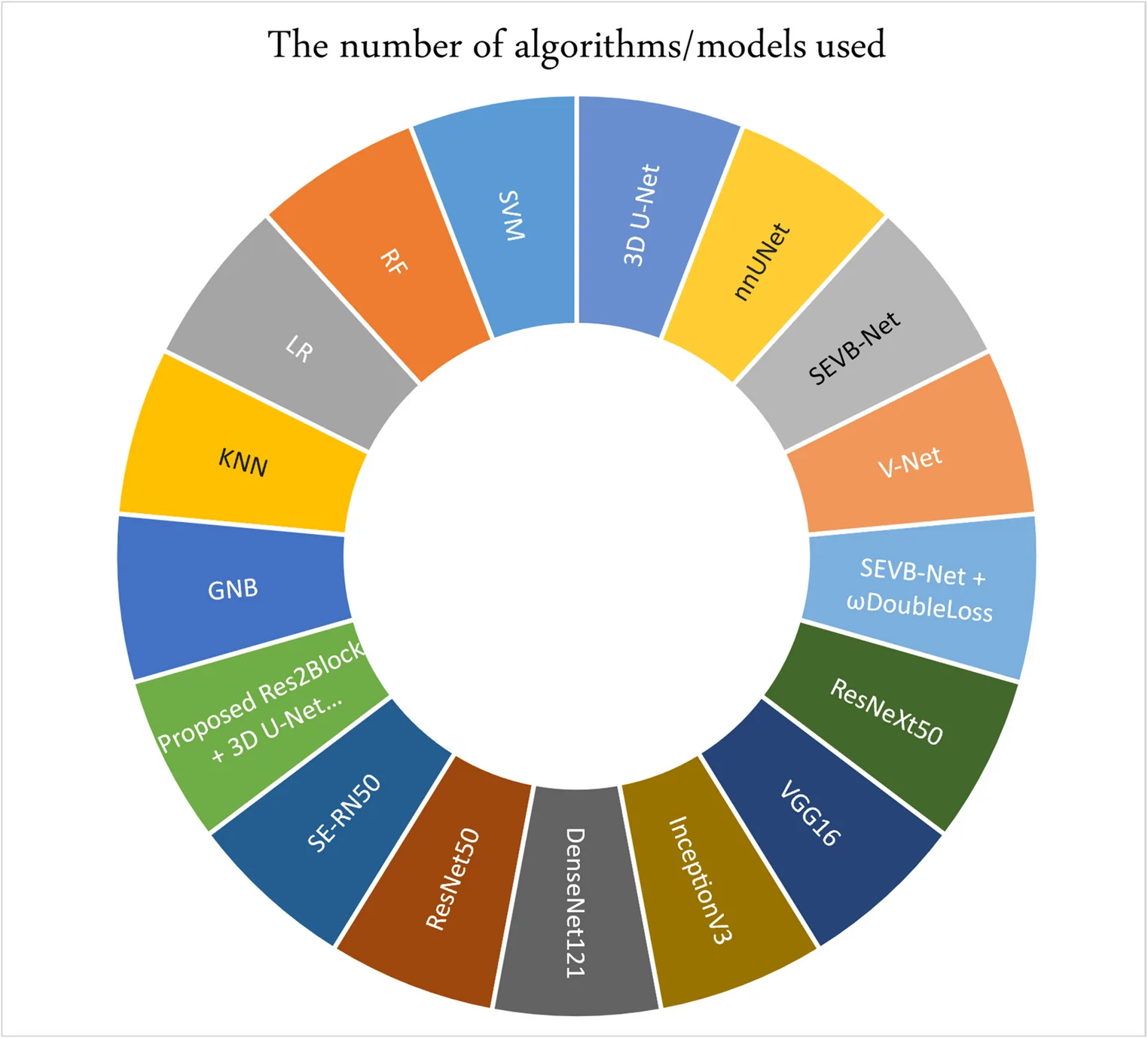
Frequency of algorithms used in the analyzed studies

In total, this review analyzed 20 AI-based models, including conventional ML classifiers and deep learning architectures. These models, such as SEVB-Net, 3D U-Net, and ResNet50, were used for trigeminal nerve segmentation and neurovascular conflict detection in trigeminal neuralgia. Deep learning models showed superior diagnostic performance, particularly in sensitivity and specificity, highlighting their potential in clinical applications (Fig. 2).

In terms of imaging modality, MRI-based sequences (including T1, T2, and MRA) were utilized in 80% (4 out of 5) of the studies. Additionally, Functional Connectivity fMRI was employed in 20% (1 out of 5) of the studies. Regarding the validation methods, cross-validation was the most common approach, used in 60% (3 out of 5) of the studies, with 5-fold cross-validation being the predominant technique. The remaining 40% (2 out of 5) studies employed a train/test split methodology for model evaluation. All five studies (100%) used imaging-derived features. Functional connectivity, on the other hand, was utilized in 20% (1 out of 5) of the studies as an additional feature (Tables 1 and 2).

Concerning radiomics methods, all studies (100%) employed automated feature extraction techniques. The most commonly used feature type was segmentation-based features, applied in 40% (2 out of 5) of the studies, followed by multi-scale and voxel-based features, which were also used in 40% (2 out of 5) of the studies (Tables 1 and 2)**.**

Finally, all studies (100%) relied on a combination of clinical diagnosis and MRI for diagnostic confirmation (Tables 1 and 2).

### Meta-analysis of outcomes

**Fig. 3:**
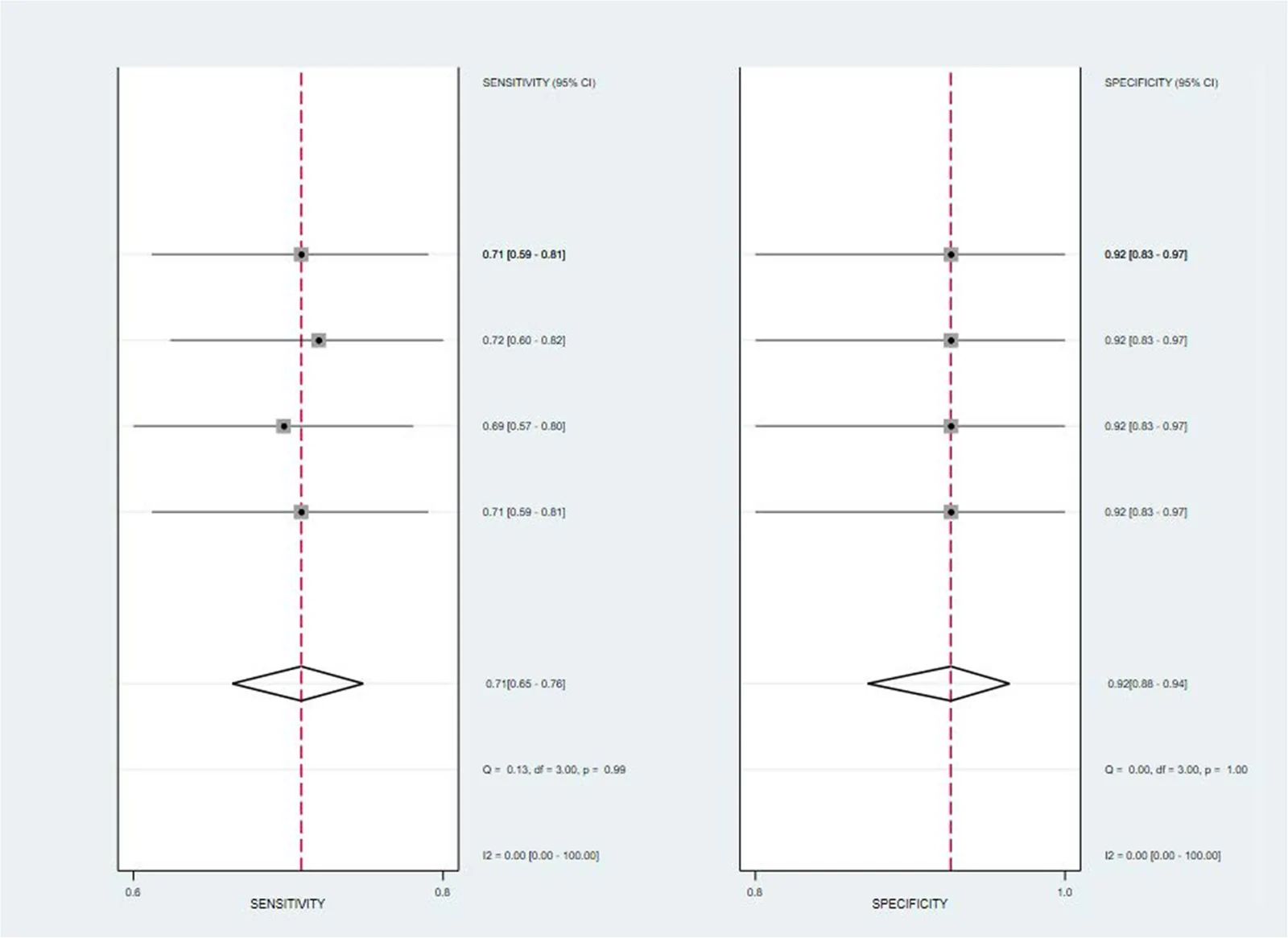
Sensitivity and specificity of ML algorithms

**Fig. 4:**
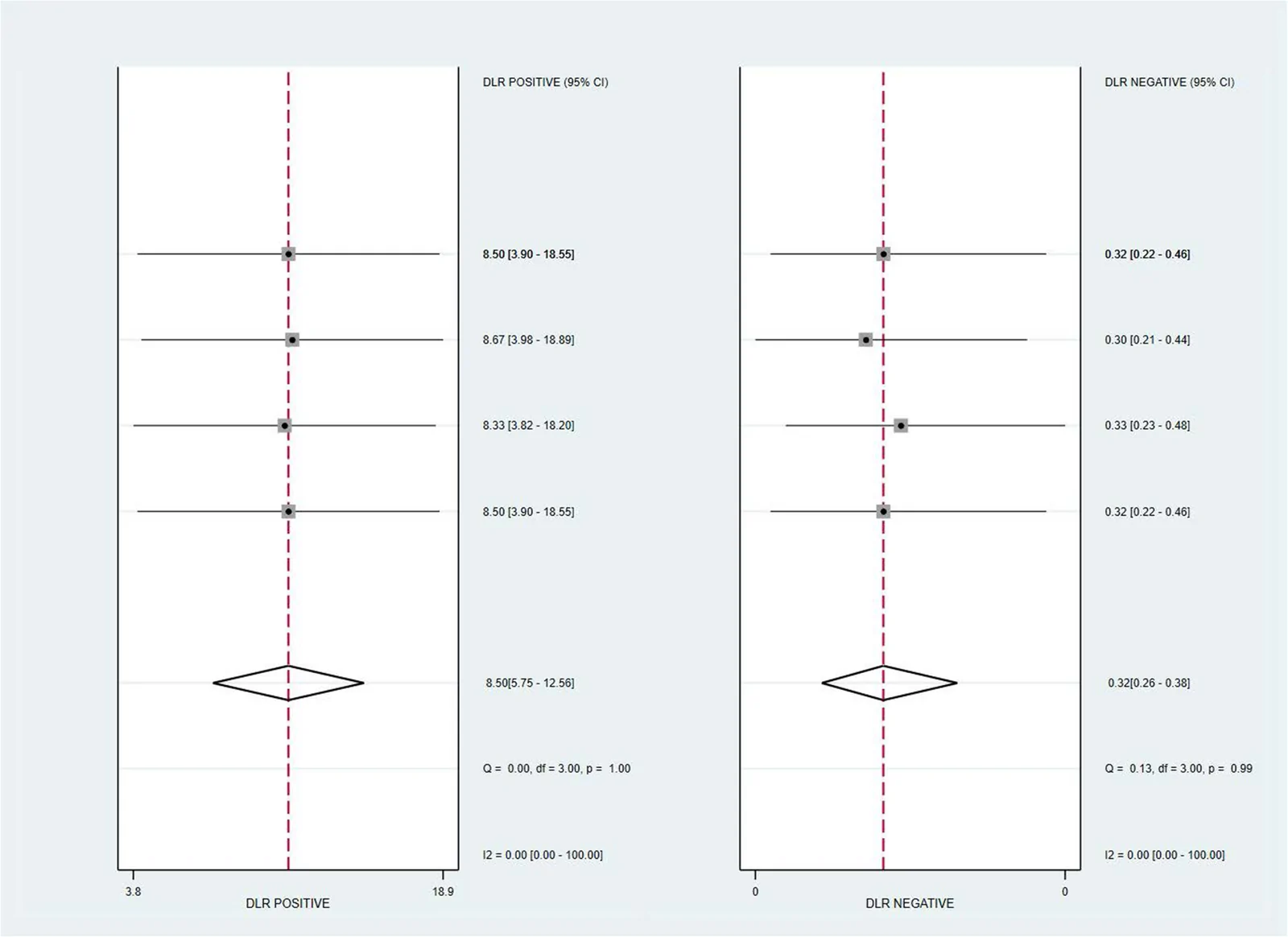
Positive and negative DLR of ML algorithms

**Fig. 5:**
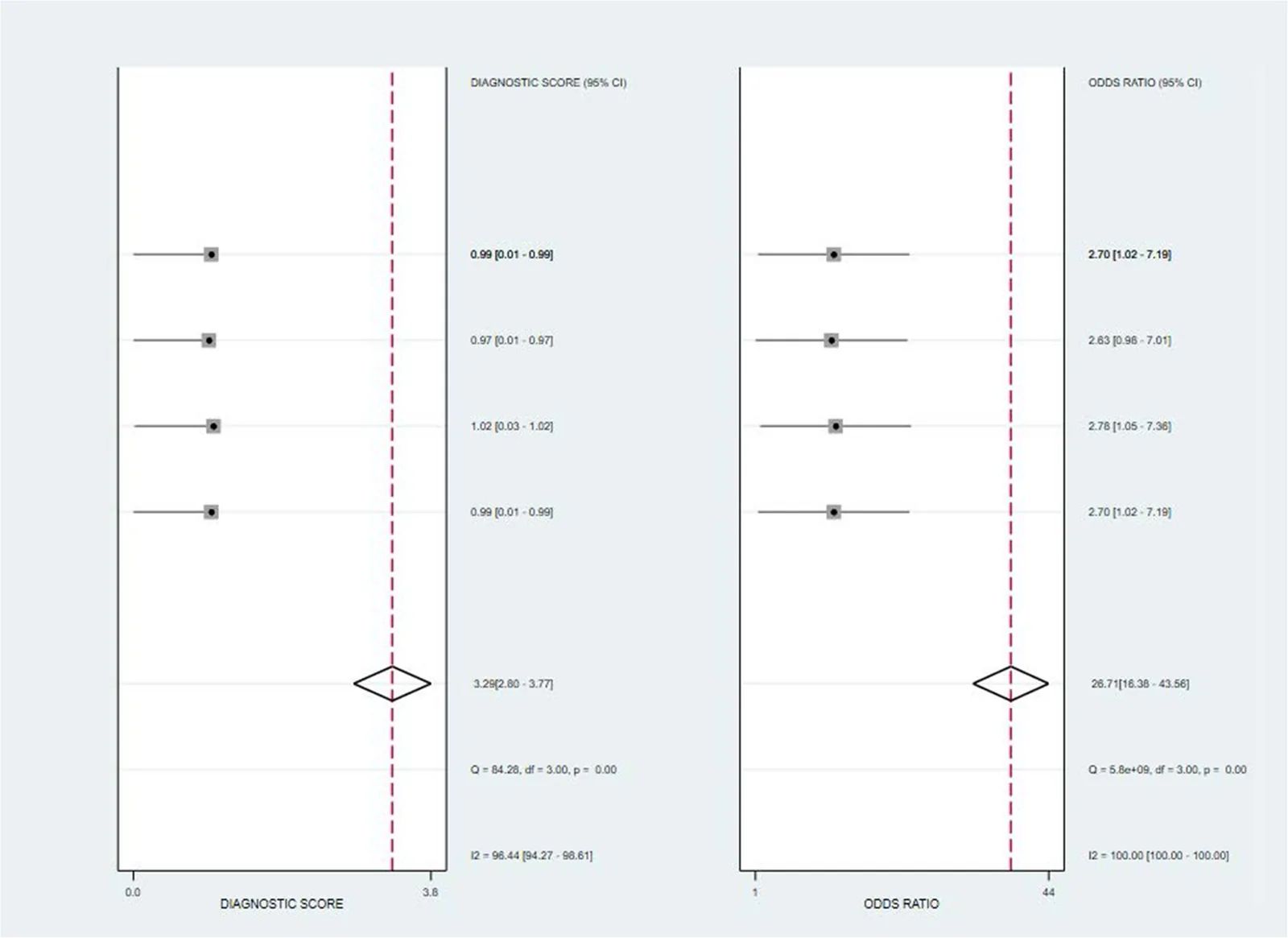
Diagnostic score and diagnostic odds ratio of ML algorithms

**Fig. 6:**
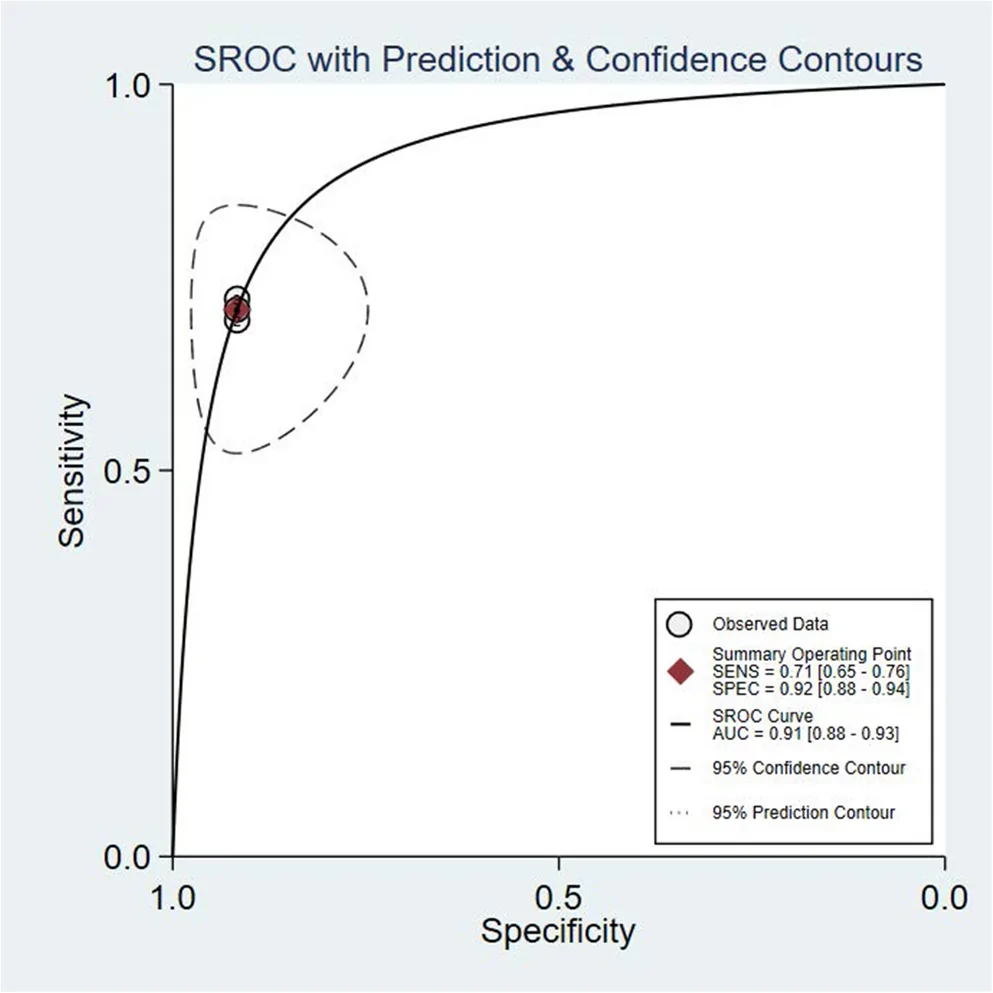
Summary receiver operator characteristic curve (SROC) of ML algorithms

The meta-analysis revealed a pooled sensitivity of 71% (95% CI: 65–76%) with substantial heterogeneity (I² = 0%). The pooled specificity was 92% (95% CI: 88–94%) with low to moderate heterogeneity (I² = 0%). The pooled positive diagnostic likelihood ratio (DLR) was 8.5 (95% CI: 5.75–12.56; I² = 0%), and the negative DLR was 0.32 (95% CI: 0.26–0.38; I² = 0%). The pooled diagnostic odds ratio (DOR) was 26.71 (95% CI: 16.38–43.56; I² = 100%), and the diagnostic score was 3.29 (95% CI: 2.8–3.77; I² = 96.44%). The summary receiver operating characteristic (SROC) curve indicated an area under the curve (AUC) of 0.91 (95% CI: 0.88–0.93) (Figs. 3, 4, 5 and 6).

### Quality assessment (PROBAST)

**Fig. 7:**
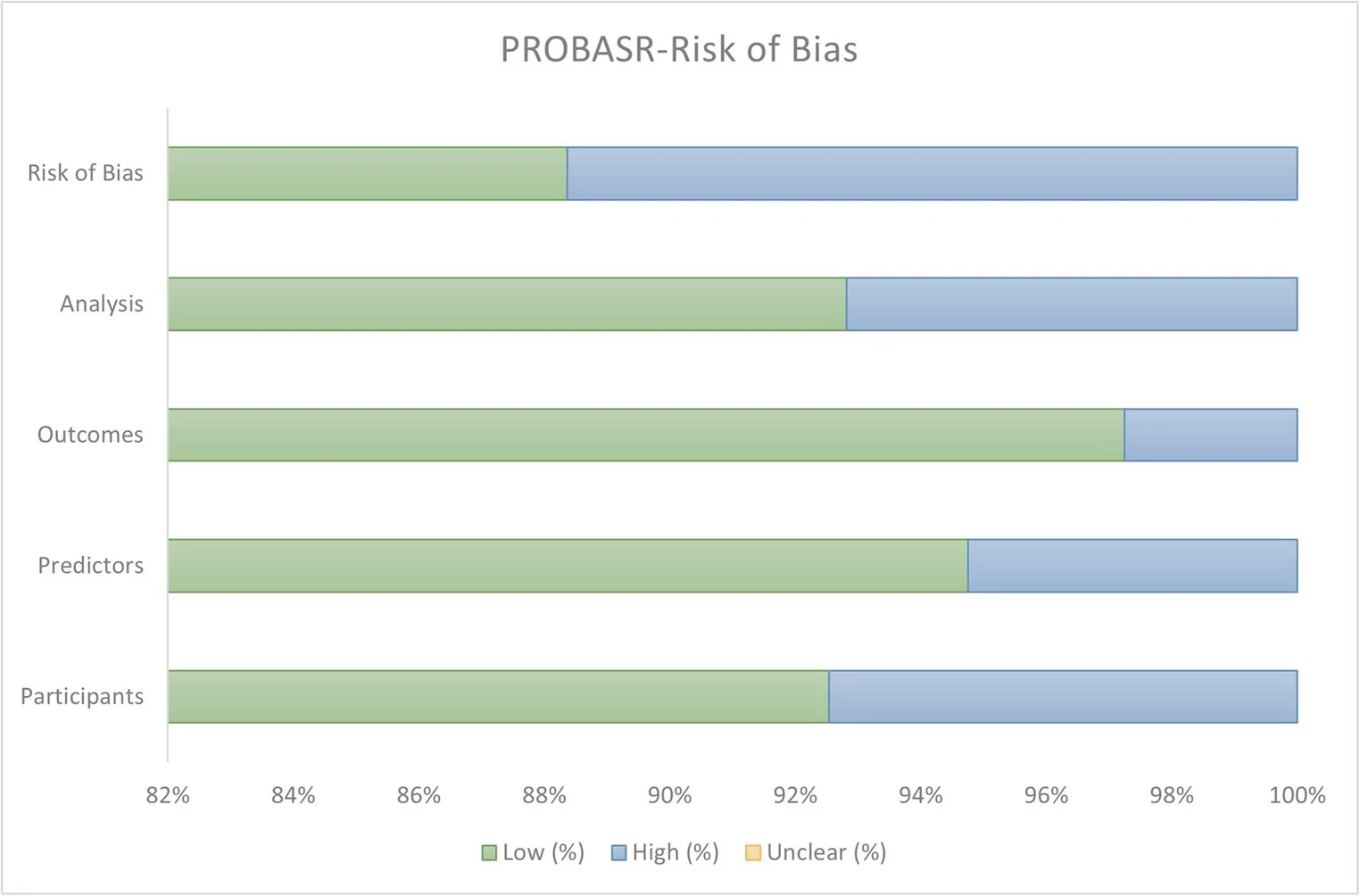
PROBAST chart showing the distribution of risk of bias across the domains of participants, predictors, outcome, and analysis

After re-evaluation using stricter PROBAST criteria, the included studies showed variable risk of bias across the participants, predictors, outcomes, and analysis domains. Although several domains were rated as low risk, concerns were identified particularly in the predictor, outcome, and analysis domains. Studies with small sample sizes, lack of independent external validation, or insufficient information regarding overfitting control were considered to have unclear or high risk in the analysis domain. In addition, studies using manual segmentation or expert imaging annotation as the reference standard were interpreted as primarily assessing technical segmentation performance rather than definitive clinical diagnostic performance, raising applicability concerns when these findings are extrapolated to clinical diagnosis or neurovascular conflict detection **(**Fig. 7**).**

**Fig. 8:**
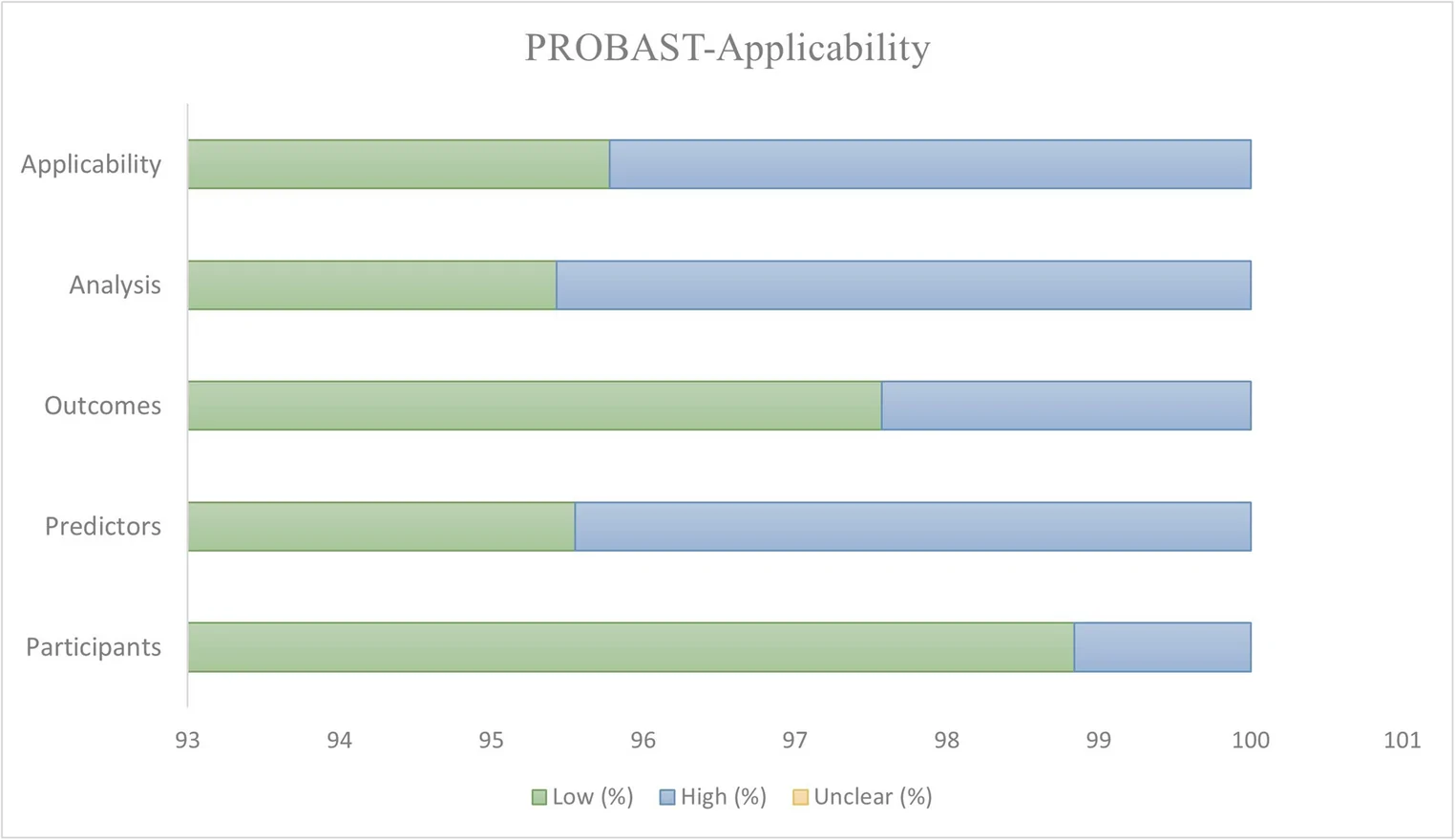
PROBAST chart illustrating the applicability assessment across the domains of participants, predictors, and outcome

Regarding applicability, 80% of studies showed low concern in the participants and outcomes domains, while 60% demonstrated low concern in the predictor domain. In 40% of studies, the applicability of predictors was unclear. No domains were identified as high-concern among the studies **(**Fig. 8**).**

## Discussion

The results of this meta-analysis demonstrate the strong potential of AI in improving the diagnostic accuracy of TN, particularly in trigeminal nerve segmentation and NVC detection. The pooled sensitivity of 71% indicates that AI models are fairly effective in detecting true TN cases, though there is room for improvement in minimizing false negatives. The pooled specificity of 92% shows that AI models excel at correctly identifying non-TN cases, reducing the risk of false positives. The positive diagnostic likelihood ratio (DLR) of 8.5 suggests that a positive AI result strongly supports the presence of TN, while the negative DLR of 0.32 indicates good performance in ruling out the condition when the test is negative. With a DOR of 26.71 and AUC of 0.91, the AI models show excellent overall diagnostic accuracy, making them valuable tools for clinical decision-making and preoperative planning in TN patients. In other neurosurgical and neurovascular applications, ML-based tools appear more mature clinically; for example, commercial ML systems for large-vessel occlusion stroke triage are already FDA-approved and increasingly used in clinical workflows, with real-world studies reporting high specificity and negative predictive value. By contrast, deep learning-based vestibular schwannoma segmentation has shown higher segmentation agreement than the current TN literature, with a pooled Dice score of approximately 0.89, but its routine clinical implementation remains limited by heterogeneity and the need for further external validation [29, 30].

Several studies have demonstrated the utility of DL techniques for segmenting the trigeminal nerve and surrounding cerebrovascular structures, which are crucial for diagnosing TN and understanding its pathophysiology. For instance, while our study focused on diagnostic metrics, many studies, such as those involving the segmentation of trigeminal nerve structures in MRI scans, have primarily evaluated model performance in terms of segmentation accuracy. Lin et al. [23]. proposed a method using DL for segmenting the trigeminal nerve and cerebrovasculature, reporting a high Dice similarity coefficient of 0.8645. Their approach offers robust segmentation, but it did not include diagnostic performance metrics such as sensitivity or specificity, which makes it challenging to directly compare with our comprehensive evaluation of diagnostic accuracy. Nonetheless, their study confirms that accurate anatomical segmentation can be a key component in improving the diagnostic and treatment outcomes for TN, especially when combined with neurovascular visualization.

Similarly, in the study by Zhang et al. [22]. the SEVB-Net model showed strong segmentation performance for the trigeminal nerve in 3D MRI volumes, with Dice coefficients ranging from 0.6070 to 0.7923. These results are promising for anatomical segmentation tasks but still fall short of the diagnostic benchmarks we report, such as the DOR of 26.71 and positive likelihood ratio (DLR) of 8.5 in our meta-analysis. While Zhang et al. focused on segmentation, our work extends the understanding by emphasizing diagnostic outcomes, critical for assessing how these models would function in a real clinical setting where accurate diagnosis is paramount.

Further, Halbert-Elliott et al [28]. used AI for segmenting both the trigeminal nerve and the surrounding vasculature. Their best-performing model, based on a SE-ResNet50 backbone, yielded a Dice score of 0.775, which is in line with the performance observed by Zhang et al [22]. However, their model, like others, focused mainly on segmentation without exploring diagnostic metrics such as sensitivity, specificity, or AUC. Our analysis, in contrast, includes a comprehensive evaluation of how AI can actually impact diagnosis, providing a more holistic view of its potential clinical applications.

While these studies primarily target segmentation, the real challenge in TN diagnosis lies in how accurately AI models can translate anatomical data into clinical decisions. Wu et al [27], for example, used functional MRI and ML to assess brain dynamics in TN patients, differentiating between subtypes based on neurovascular compression. Though not directly related to segmentation, their work demonstrates that AI can be useful not only in structural imaging but also in understanding functional differences in TN patients. However, their approach did not directly assess diagnostic accuracy in the same way our meta-analysis did, which highlights the gap between AI’s potential for enhancing structural understanding and its actual clinical diagnostic utility [23, 24].

The key takeaway from comparing these studies with our findings is that while there has been significant progress in using AI for segmentation tasks, fewer studies have integrated diagnostic performance measures such as sensitivity, specificity, and AUC. These are critical for understanding how AI models can be deployed in clinical settings to improve diagnosis and treatment planning. In our meta-analysis, we found a high specificity (92%) and a high AUC of 0.91, indicating that AI models, when designed and validated with diagnostic metrics in mind, can perform well not only in segmentation but also in clinical decision-making.

### Limitation

Although AI-based models for TN diagnosis have made significant strides in improving the accuracy of segmentation and neurovascular conflict (NVC) detection, several limitations persist. One of the main challenges is that many of the studies in this field focus primarily on segmentation accuracy without considering broader clinical metrics, such as diagnostic sensitivity, specificity, and AUC. While segmentation is a critical first step in identifying TN and NVC, it is essential for AI models to be evaluated in the context of their diagnostic utility in real-world clinical practice. Our meta-analysis addresses this gap by evaluating models on diagnostic performance, providing a more comprehensive view of their clinical potential. The risk-of-bias assessment also has important implications for interpretation. Some included studies used manual segmentation or expert imaging annotation as the reference standard, which is suitable for evaluating technical segmentation accuracy but does not necessarily represent a true clinical endpoint. This may introduce circularity and could inflate apparent model performance, particularly when external clinical validation or intraoperative confirmation is absent. In addition, small sample sizes, limited reporting of model calibration, lack of independent external validation, and potential overfitting remain important methodological concerns in the available literature. Additionally, many included studies relied on internal validation methods such as cross-validation or train/test splits. These methods may produce slightly optimistic performance estimates because the results are not fully independent. Our meta-analysis used the MIDAS package to pool sensitivity and specificity, which provides robust estimates for diagnostic performance, but this limitation should be considered when interpreting the findings.

### Future direction

To further advance AI models in TN diagnosis, future research should focus on improving the integration of these models into clinical workflows with an emphasis on diagnostic performance. In addition to segmentation accuracy, models should be evaluated based on metrics like sensitivity, specificity, and AUC to assess their clinical utility.

Combining structural imaging with functional data, such as fMRI, could enhance AI models’ ability to capture the complexity of TN and its neurovascular mechanisms. Furthermore, developing models that can adapt to various imaging protocols and diverse patient populations will be essential for their broader clinical application.

Another limitation of current AI approaches is the generalizability of these models across different clinical settings. Many studies rely on specific datasets, image resolutions, or types of imaging equipment, which may limit the robustness of AI models when applied to a broader range of patients or medical institutions. The variability in patient demographics, imaging protocols, and data quality can impact the model’s accuracy and performance in real-world applications, highlighting the need for more diverse and representative datasets in future research.

## Conclusion

In conclusion, while AI-based models for TN diagnosis have shown significant promise in segmentation and neurovascular conflict detection, our meta-analysis highlights the importance of incorporating diagnostic metrics into the evaluation of these models. By focusing on sensitivity, specificity, and AUC, our study provides a more comprehensive understanding of how AI can improve TN diagnosis in clinical practice. The comparison with existing literature underscores the need for further research to refine AI models, ensure their clinical applicability, and ultimately enhance patient care in the management of TN.

## Supplementary Information

Below is the link to the electronic supplementary material.

Supplementary File 1 (DOCX 17.2 KB)

## Data Availability

No datasets were generated or analysed during the current study.

## Declarations

### Originality and exclusivity

Not applicable. The manuscript is original and has not been published or submitted elsewhere.

### Compliance with instructions

The authors confirm the manuscript follows all journal guidelines.

### AI Usage Statement

During manuscript preparation, the authors used Anthropic's Claude (Opus 4.7) and OpenAI's ChatGPT (GPT-5) for language refinement, editorial review, and drafting of prose summarizing author-generated statistical output. These tools were not used to design the study, conduct or interpret analyses, generate data, or produce citations. All analyses were performed by the authors in STATA (v17). All AI-assisted text was reviewed and verified by the authors, who take full responsibility for the manuscript's content. No confidential patient information was shared.
