# Supplementary material for: Artificial intelligence in trigeminal neuralgia: trigeminal nerve segmentation and neurovascular conflict detection: a systematic review and meta-analysis
Source: Neurosurg Rev. 2026 Sep 14;49(1):566. doi: 10.1007/s10143-026-04486-5 (PMC13574729; doi:10.1007/s10143-026-04486-5)
Supplement: Supplementary file 1 [file 10143_2026_4486_MOESM1_ESM.docx]

| **Table S1.** The search strategy of electronic databases | | |
| --- | --- | --- |
| **Database** | **Search Strategy** | **Number** |
| **PubMed** | #1 "facial neuralgia"[MeSH Terms] OR ("facial"[All Fields] AND "neuralgia"[All Fields]) OR "facial neuralgia"[All Fields] OR ("trigeminal neuralgia"[MeSH Terms] OR ("trigeminal"[All Fields] AND "neuralgia"[All Fields]) OR "trigeminal neuralgia"[All Fields]) OR ("trigeminal neuralgia"[MeSH Terms] OR ("trigeminal"[All Fields] AND "neuralgia"[All Fields]) OR "trigeminal neuralgia"[All Fields] OR ("tic"[All Fields] AND "douloureux"[All Fields]) OR "tic douloureux"[All Fields]) OR ("trigeminal neuralgia"[MeSH Terms] OR ("trigeminal"[All Fields] AND "neuralgia"[All Fields]) OR "trigeminal neuralgia"[All Fields] OR ("trifacial"[All Fields] AND "neuralgia"[All Fields]) OR "trifacial neuralgia"[All Fields]) OR ("facial neuralgia"[MeSH Terms] OR ("facial"[All Fields] AND "neuralgia"[All Fields]) OR "facial neuralgia"[All Fields])  #2 "machine learning"[Title/Abstract] OR "deep learning"[Title/Abstract] OR "artificial intelligence"[Title/Abstract] OR "neural network"[Title/Abstract] OR "supervised learning"[Title/Abstract] OR "predictive model"[Title/Abstract] OR "neural networks computer"[Title/Abstract] OR "computer vision"[Title/Abstract] OR "artificial neural networks"[Title/Abstract] OR "conventional neural network"[Title/Abstract] OR "reinforcement learning"[Title/Abstract] OR "transfer learning"[Title/Abstract] OR ("natural language processing"[MeSH Terms] OR ("natural"[All Fields] AND "language"[All Fields] AND "processing"[All Fields]) OR "natural language processing"[All Fields]) OR (("drugs, generic"[MeSH Terms] OR ("drugs"[All Fields] AND "generic"[All Fields]) OR "generic drugs"[All Fields] OR "generic"[All Fields] OR "family characteristics"[MeSH Terms] OR ("family"[All Fields] AND "characteristics"[All Fields]) OR "family characteristics"[All Fields] OR "generation"[All Fields] OR "generations"[All Fields] OR "general"[All Fields] OR "general s"[All Fields] OR "generalisability"[All Fields] OR "generalisable"[All Fields] OR "generalisation"[All Fields] OR "generalization, psychological"[MeSH Terms] OR ("generalization"[All Fields] AND "psychological"[All Fields]) OR "psychological generalization"[All Fields] OR "generalization"[All Fields] OR "generalisations"[All Fields] OR "generalise"[All Fields] OR "generalised"[All Fields] OR "generalises"[All Fields] OR "generalisibility"[All Fields] OR "generalising"[All Fields] OR "generalities"[All Fields] OR "generality"[All Fields] OR "generalizability"[All Fields] OR "generalizable"[All Fields] OR "generalizations"[All Fields] OR "generalize"[All Fields] OR "generalized"[All Fields] OR "generalizes"[All Fields] OR "generalizing"[All Fields] OR "generally"[All Fields] OR "generals"[All Fields] OR "generate"[All Fields] OR "generated"[All Fields] OR "generates"[All Fields] OR "generating"[All Fields] OR "generation s"[All Fields] OR "generational"[All Fields] OR "generative"[All Fields] OR "generatively"[All Fields] OR "generativity"[All Fields] OR "generator"[All Fields] OR "generator s"[All Fields] OR "generators"[All Fields] OR "generically"[All Fields] OR "genericity"[All Fields] OR "generics"[All Fields]) AND ("model"[All Fields] OR "model s"[All Fields] OR "modeled"[All Fields] OR "modeler"[All Fields] OR "modeler s"[All Fields] OR "modelers"[All Fields] OR "modeling"[All Fields] OR "modelings"[All Fields] OR "modelization"[All Fields] OR "modelizations"[All Fields] OR "modelize"[All Fields] OR "modelized"[All Fields] OR "modelled"[All Fields] OR "modeller"[All Fields] OR "modellers"[All Fields] OR "modelling"[All Fields] OR "modellings"[All Fields] OR "models"[All Fields])) OR "recurrent neural networks"[Title/Abstract] OR "linear regression"[Title/Abstract] OR "random forests"[Title/Abstract] OR "gradient boosting machines"[Title/Abstract]  #1 AND #2: | 248 |
| **Embase** | #1 'machine learning'/exp OR 'deep learning'/exp OR 'artificial intelligence'/exp  #2 Trigeminal Neuralgia /exp  **#1 AND #2 :** | 38 |
| **Scopus** | ( TITLE-ABS-KEY ( Trigeminal Neuralgia ) ) AND ( ( TITLE-ABS-KEY ( machine AND learning ) OR TITLE-ABS-KEY ( deep AND learning ) OR TITLE-ABS-KEY ( artificial AND intelligence ) ) ) | 58 |
| **Web of Science** | #1 TS=(("trigeminal neuralgia" OR "tic douloureux" OR "trifacial neuralgia"  OR "facial neuralgia" OR "trigeminal neuropathic pain" OR "trigeminal nerve disease*"  OR "trigeminal nerve disorder*" OR "fifth cranial nerve neuralgia" OR "cranial neuralgia"))  #2 (((((ALL=(machin learning )) OR ALL=(deep learning)) OR ALL=(artificial intelligence )) OR TS=(conventional neural network)) OR TS=(transfer of learning)) OR TS=(transfer of learning)  #3 ((TS=(Survive)) OR TS=(Survival)) OR TS=(Surviving)  **#1 AND #2 AND #3 :** | 83 |
| **Google scholar** | ("machine learning" OR "deep learning" OR artificial intelligent" ) AND (" Trigeminal Neuralgia" OR "facial neuralgia OR “ trigeminal neuropathic pain”") | First 10 pages (100) |

*22/4/ 2025. 2.35 P.M*

Total: 527, duplicates, 63, for screen: 464
